# Artificial intelligence in mammography: a systematic review of the external validation

**DOI:** 10.61622/rbgo/2024rbgo71

**Published:** 2024-09-04

**Authors:** Paulo Eduardo Souza Castelo Branco, Adriane Helena Silva Franco, Amanda Prates de Oliveira, Isabela Maurício Costa Carneiro, Luciana Maurício Costa de Carvalho, Jonathan Igor Nunes de Souza, Danniel Rodrigo Leandro, Eduardo Batista Cândido

**Affiliations:** 1 Faculdade de Medicina Faculdade de Minas Belo Horizonte MG Brazil Faculdade de Medicina, Faculdade de Minas, Belo Horizonte, MG, Brazil.; 2 Faculdade de Medicina Universidade Federal dos Vales do Jequitinhonha e Mucuri Diamantina MG Brazil Faculdade de Medicina, Universidade Federal dos Vales do Jequitinhonha e Mucuri, Diamantina, MG, Brazil.; 3 Universidade Federal de Minas Gerais Belo Horizonte MG Brazil Universidade Federal de Minas Gerais, Belo Horizonte, MG, Brazil.

**Keywords:** Artificial Intelligence, Mammography, Deep learning, Breast neoplasms, Machine learning

## Abstract

**Objective:**

To conduct a systematic review of external validation studies on the use of different Artificial Intelligence algorithms in breast cancer screening with mammography.

**Data source:**

Our systematic review was conducted and reported following the PRISMA statement, using the PubMed, EMBASE, and Cochrane databases with the search terms “Artificial Intelligence,” “Mammography,” and their respective MeSH terms. We filtered publications from the past ten years (2014 – 2024) and in English.

**Study selection:**

A total of 1,878 articles were found in the databases used in the research. After removing duplicates (373) and excluding those that did not address our PICO question (1,475), 30 studies were included in this work.

**Data collection:**

The data from the studies were collected independently by five authors, and it was subsequently synthesized based on sample data, location, year, and their main results in terms of AUC, sensitivity, and specificity.

**Data synthesis:**

It was demonstrated that the Area Under the ROC Curve (AUC) and sensitivity were similar to those of radiologists when using independent Artificial Intelligence. When used in conjunction with radiologists, statistically higher accuracy in mammogram evaluation was reported compared to the assessment by radiologists alone.

**Conclusion:**

AI algorithms have emerged as a means to complement and enhance the performance and accuracy of radiologists. They also assist less experienced professionals in detecting possible lesions. Furthermore, this tool can be used to complement and improve the analyses conducted by medical professionals.

## Introduction

Breast cancer is the most common neoplasm in women worldwide. In Brazil, this scenario is no different, with higher incidence rates in the Southeastern and Midwest regions of the country, areas with higher Human Development Index, life expectancy, later pregnancies, and fewer children.^1^ It is estimated that there will be more than 73,000 new cases of breast cancer per year in Brazil in 2024 and 2025.^2^

Mammography, a radiological exam that involves taking images in cranio-caudal (CC) and medio-lateral-oblique (MLO) of each breast of the woman, is the basis of breast cancer screening. The Breast Imaging-Reporting and Data System (BI-RADS) is a globally accepted method for naming findings in breast imaging exams. It is a standardized nomenclature system that classifies the risk of malignancy of radiological findings, including situations where there is no finding or when the findings are certainly benign. However, although mammography is widely regarded as the gold standard for finding breast cancer, screening programs are always being discussed in light of new technology to cut back on wasteful biopsies and treatments, incorrect diagnoses, and enhance early cancer detection.^3^

To address limitations in mammography screening, artificial intelligence (AI)-assisted diagnostic models were launched as a support tool in the 1990s. Deep Learning techniques analyze tissue properties using complex algorithms and image processing technologies, helping medical professionals interpret radiological scans more accurately and expediting interpretation time. Additionally, AI systems can improve screening sensitivity and support general practitioners in correctly interpreting mammograms.^3-5^

The objective of this article is to conduct an organized evaluation of research that has provided external validation for the use of different AI algorithms in breast cancer screening with mammography. Studies that assessed the algorithm in an entirely distinct environment from that used for its creation were chosen because external validation of research is reliant on the efficacy of that study being relevant to other populations.^6^

## Methods

Our systematic review was conducted and reported following the Preferred Reporting Items for Systematic Reviews and Meta-Analyses (PRISMA) statement.^7^ Our review protocol was registered in the International Prospective Register of Systematic Reviews (PROSPERO, ID: CRD42023461935).^8^ The terms “Artificial Intelligence” and “Mammography,” along with their respective Medical Subject Headings (MeSH), were used to search the PubMed, Cochrane, and EMBASE databases (details of the search strategy are shown in (Chart 1).

**Chart 1:** Search strategy

Databases	Terms
PubMed	((“Artificial Intelligence”[MeSH Terms] OR “Artificial Intelligence”[Title/Abstract]) AND (“mammography”[MeSH Terms] OR “mammographic screening”[Title/Abstract] OR “digital breast tomosynthesis”[Title/Abstract] OR “digital mammography”[Title/Abstract])) AND (y_10[Filter])
Cochrane	“Artificial Intelligence” AND “mammography” OR “mammographic screening” OR “digital breast tomosynthesis” OR “digital mammography” AND (y_10[Filter])
EMBASE	‘Artificial intelligence’ AND (‘mammography’ OR ‘mammographic screening’ OR ‘digital breast tomosynthesis’ OR ‘digital mammography’) AND [2014-2024]/py

Description - the terms and their respective MeSH used to search for articles in the PubMed, Cochrane, and EMBASE databases

**Figure 1:**
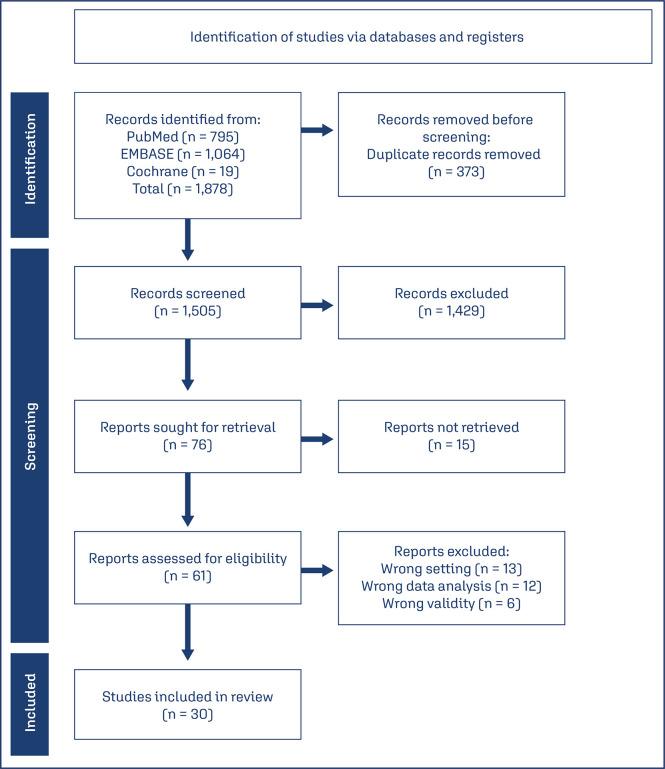
Study selection process Description - Based on the PRISMA strategy and the PICO question, a schematic representation of the article selection procedure used for this systematic review is provided

Publications from the past ten years (01.2014 – 04.2024) and written in English were filtered. Studies that performed external validation of AI algorithms for mammography-based breast cancer detection (either by themselves or in conjunction with radiologists) were included. We excluded studies that provided algorithm training details, evaluated future cancer risk using AI, internally validated the algorithm (representing true results for the same sample used in the algorithm’s development), or externally evaluated algorithms using public image databases used for training and developing various Deep Learning models. Studies that provided both internal and external validation were only taken into account for the outcomes of the external validation. The systematic review also excluded studies that did not report findings based on accuracy, sensitivity, specificity, and/or area under the ROC curve (AUC). Our method, based on the PRISMA strategy, can be observed in the scheme represented in figure 1.

In the databases used for the study, a total of 1,878 articles were discovered; 373 duplicate studies were subsequently eliminated. Based on reading the article titles and abstracts, only 76 articles remained after studies were excluded that did not address the PICO question proposed for this study. Fifteen of these articles were unavailable for reading at no cost or in author-accessible databases. 30 studies that matched the goal of this work were chosen after the 61 articles were reviewed. All the titles and abstracts obtained from the literature research were independently reviewed by the two authors for inclusion and exclusion criteria, with disagreements settled by consensus. Five authors independently gathered study data, which was then combined based on sample data, study year, location, and the main findings in terms of AUC, sensitivity, and specificity. Using the Quality Assessment of Diagnostic Accuracy Studies-2 (QUADAS-2) tool, the studies’ overall methodological quality was independently evaluated.^9^

## Results

All 30 studies included in this systematic review were conducted with external validation (internal validation data were removed) of Artificial Intelligence algorithms (Convolutional Neural Network and Design Automation Conference, methods integrated with Deep Learning) used for breast cancer screening (Chart 2)^5,10-38^ presents all articles with their sample size, country, type of study, and type of AI assessment). Among those, 18 studies evaluated the algorithm as an independent reader, 8 studies assessed the precision of independent radiologists and radiologists in association with AI, and five studies examined both scenarios. Of the studies that evaluated both scenarios, Rodríguez-Ruiz et al.^10^observed an improvement in the performance of radiologists with the support of Artificial Intelligence (Δ 0.02; p = 0.002), but when independent AI was evaluated, there was no apparent distinction in the AUC between it and radiologists’ performance (Δ 0.02; p = 0.33) (Chart 2). ^5,10-38^

**Chart 2:** Studies selected in this systematic review

Resources	Sample size*	Nation	Study types**	Type of AI assessment***
Lee et al. (2022)^(4)^	200	South Korea	Retrospective	Radiologist without AI vs. Radiologist with AI
Zhou et al. (2023)^(5)^	880	China	Retrospective	Radiologist without AI vs. Radiologist with AI
Rodríguez-Ruiz et al. (2019)^(10)^	240	USA and Netherlands	Retrospective	Radiologist vs. AI AND Radiologist without AI vs. Radiologist with AI
Sun et al. (2021)^(36)^	200	China	Retrospective	Radiologist vs. AI AND Radiologist without AI vs. Radiologist with AI
	5,746	China	Prospective	Radiologist with AI
Lee et al. (2024)^(34)^	2,061	South Korea	Retrospective	Radiologist vs. AI AND Radiologist without AI vs. Radiologist with AI
Yala et al. (2019)^(11)^	26,540	USA	Retrospective	Radiologist vs. AI
Rodríguez-Ruiz et al. (2019)^(12)^	2,652	Netherlands	Retrospective	Radiologist vs. AI
Liao et al. (2023)^(35)^	460	China	Retrospective	Radiologist vs. AI
Leibig et al. (2022)^(13)^	82,851	Germany	Retrospective	Radiologist vs. AI
Marinovich et al. (2023)^(14)^	108,970	Australia	Retrospective	Radiologist vs. AI
Akselrod-Ballin et al. (2019)^(15)^	2,548	Israel	Retrospective	Radiologist vs. AI
Lauritzen et al. (2022)^(17)^	114,421	Denmark	Retrospective	Radiologist vs. AI
Salim et al. (2020)^(18)^	8,805	Sweden	Retrospective	Radiologist vs. AI
Sharma et al. (2023)^(19)^	275,900	UK and Hungary	Retrospective	Radiologist vs. AI
Yirgin et al. (2022)^(20)^	22,621	Türkiye	Retrospective	Radiologist vs. AI
Bao et al. (2023)^(21)^	643	China	Retrospective	Radiologist without AI vs. Radiologist with AI
Dang et al. (2022)^(22)^	314	France	Retrospective	Radiologist without AI vs. Radiologist with AI
Watanabe et al. (2019)^(23)^	122	USA	Retrospective	Radiologist without AI vs. Radiologist with AI
Kim et al. (2022)^(24)^	793	South Korea	Retrospective	Radiologist without AI vs. Radiologist with AI
Pacilè et al. (2020)^(25)^	240	France	Retrospective	Radiologist without AI vs. Radiologist with AI
Romero-Martín et al. (2022)^(26)^	15,999	Spain	Retrospective	Radiologist vs. AI
Hsu et al. (2022)^(27)^	37,317	USA	Retrospective	Radiologist without AI vs. Radiologist with AI
Liu et al. (2021)^(28)^	51	China	Retrospective	Radiologist vs. AI
Sasaki et al. (2020)^(29)^	310	Japan	Retrospective	Radiologist vs. AI
Al-Bazzaz et al. (2024)^(30)^	758	Sweden	Retrospective	Radiologist vs. AI AND Radiologist without AI vs. Radiologist with AI
Do et al. (2021)^(31)^	435	South Korea	Retrospective	Radiologist vs. AI
Elhakim et al. (2023)^(32)^	257,671	Denmark	Retrospective	Radiologist vs. AI AND Radiologist without AI vs. Radiologist with AI
Kühl et al. (2024)^(33)^	249,402	Denmark	Retrospective	Radiologist vs. AI
Waugh et al. (2024)^(37)^	7,533	Australia	Retrospective	Radiologist vs. AI
Yoon et al. (2023)^(38)^	6,499	South Korea	Retrospective	Radiologist vs. AI

Description - The table presents all articles with their sample size, country, type of study, and type of AI assessment. *Sample size - number of mammograms analyzed; **Study types - retrospective OR prospective; ***Type of AI assessment - radiologist vs. AI (independent reader) OR radiologist without AI vs. radiologist with AI (combined reader)

Sun et al.^36^observed a statistically significant variation greater than Rodríguez-Ruiz et al.^10^in the performance of radiologists with AI (Δ 0.047; p = 0.005), which differs from the findings of Lee et al.^34^in which there was a variation of 0.134, which was not statistically significant (Δ 0.134; p = 0.146). However, all these studies, when evaluated independently (Radiologist vs. AI), showed non-statistically different variations in AUC (p > 0.05). The included studies were published between 2018 and 2024, and 29 were retrospective analyses of mammograms conducted between 2009 and 2022. These tests were executed in the United States, Europe, the United Kingdom, Australia, China, the Middle East, Japan, and South Korea. Furthermore, one of the studies was carried out by Sun et al.^36^in Beijing, China, in which they evaluated an AI system retrospectively (independently and associated with a radiologist) and prospectively. The prospective evaluation was carried out in six hospital centers in China, where the performance of radiologists with AI was evaluated, however, it was not compared with the performance of radiologists without AI. In an analysis of 5,746 mammograms, the sensitivity, specificity, and AUC of radiologists with AI were 0.943, 0.98, and 0.967, respectively. In chart 3,^13-38^the independently examined Deep Learning algorithms’ performance (AUC, sensitivity, and specificity) is shown. One of the retrospective studies was done by Yala et al.^11^ in Boston and showed an AUC of 0.82 for AI during their test study. Additionally, compared to radiologists, it demonstrated a statistically significant improvement in specificity ( Δ 0.007; p = 0.002) and non-inferior sensitivity ( Δ -0.005; p < 0.001).

**Chart 3:** Independent artificial intelligence performance

Researches	Radiologist performance accuracy			AI performance accuracy		
	AUC	Sensitivity	Specificity	AUC	Sensitivity	Specificity
Marinovich et al. (2023)^(14)^	0.93	0.68	0.97	0.83	0.67	0.81
Sharma et al. (2023)^(19)^	NR	0.885-0.888	0.947-0.979	NR	0.723-0.849	0.893-0.962
Lauritzen et al. (2022)^(17)^	NR	0.708	0.981	0.91	0.697	0.986
Yirgin et al. (2022)^(20)^	NR	0.673	NR	0.853	0.728	0.883
Leibig et al. (2022)^(13)^	NR	0.872	0.934	0.951	0.846	0.913
Romero-Martín et al. (2022)^(26)^	NR	0.584	0.8	0.93	0.628	0.8
Salim et al. (2020)^(18)^	NR	0.774	0.966	0.956	0.819	0.966
Sasaki et al. (2020)^(29)^	0.816	0.89	0.86	0.706	0.85	0.67
Yala et al. (2019)^(11)^	NR	0.906	0.936	0.82	NR	NR
Akselrod-Ballin et al. (2019)^(15)^	NR	NR	NR	0.91	0.87	0.773
Rodríguez-Ruiz et al. (2019)^(12)^	0.814	NR	NR	0.84	NR	NR
Rodríguez-Ruiz et al. (2019)^(10)^	0.85	0.83	0.77	0.89	NR	NR
Liu et al. (2021)^(28)^	0.92	0.912	0.892	0.91	0.853	0.919
Al-Bazzaz et al. (2024)^(30)^	NR	0.64	0.96	NR	0.69	0.96
Do et al. (2021)^(31)^	0.710-0.722	0.537-0.544	0.85-0.892	0.718-0.745	0.591-0.691	0.69-0.782
Elhakim et al. (2023)^(32)^	NR	0.637	0.978	NR	0.586	0.965
Kühl et al. (2024)^(33)^	0.859	0.74	0.978	0.914	0.626	0.975
Lee et al. (2024)^(34)^	0.71	0.5	0.919	0.723	0.5	0.946
Liao et al. (2023)^(35)^	0.564-0.904	0.323-0.862	0.790-0.947	0.778	0.646	0.909
Sun et al. (2021)^(36)^	0.805	0.687	0.82	0.835	0.814	0.785
Waugh et al. (2024)^(37)^	NR	0.946-1.0	0.911	NR	0.94	0.901
Yoon et al. (2023)^(38)^	NR	0.679	0.969	NR	0.821	0.903

Description - The table shows the accuracy data for Deep Learning algorithms that have been evaluated independently. These data originated from the relevant studies; AUC - Area Under the ROC Curve; NR - not reported

Some studies have demonstrated higher AUC values than radiologists who don’t use Deep Learning algorithms, such as the study by Rodríguez-Ruiz et al.,^12^ which demonstrated a difference of 0.026 compared to the average of 101 radiologists in the United Kingdom (although always lower than the AUC of the best radiologist). Liao et al.^35^demonstrated a variation of 0.214 in the AUC of the AI algorithm compared to a junior radiologist with 5 years of experience, whereas when compared to a senior breast specialist radiologist with more than 20 years of experience, the AI presented results much lower (AUC AI: 0.778 vs. senior: 0.904). In contrast to the best radiologists, they additionally found that their sensitivity and specificity were lower. AUC values are consistently getting better when compared to older algorithms, but they continue to rise closer to the values of the top radiologists.^13,14^ When comparing artificial intelligence’s ability to detect interval cancers to that of radiologists, it was found that the algorithms detected a substantial number of interval cancers that radiologists missed. Depending on the study, this type of cancer’s sensitivity and AUC ranged from 0.29 to 0.48 and 0.67 to 0.74, respectively.^14-20^ Studies contrasting radiologists’ accuracy to that associated with AI found a significant improvement in AUC (Chart 4). Additionally, sensitivity increased significantly, while specificity statistically did not change. It was found that the improvement in performance and accuracy was more pronounced in radiologists with fewer years of professional experience.^4,5,10,21-23^

**Chart 4:** Performance of radiologists and radiologists with AI

Resources	AUC			
	Radiologist without AI	Radiologist with AI	Variation	p-value
Rodríguez-Ruiz et al. (2019)^(10)^	0.87	0.89	0.02	0.002
Watanabe et al. (2019)^(23)^	0.759	0.814	0.055	< 0.01
Pacilè et al. (2020)^(25)^	0.769	0.797	0.028	0.035
Bao et al. (2023)^(21)^	0.84	0.91	0.07	< 0.01
Kim et al. (2022)^(24)^	0.79	0.89	0.1	< 0.001
Dang et al. (2022)^(22)^	0.739	0.773	0.034	0.004
Lee et al. (2022)^(4)^	0.684	0.833	0.149	< 0.001
Zhou et al. (2023)^(5)^	0.803	0.879	0.076	< 0.001
Hsu et al. (2022)^(27)^*	NR	0.935	NR	NR
Lee et al. (2024)^(34)^	0.71	0.844	0.134	0.146
Sun et al. (2021)^(36)^	0.805	0.852	0.047	0.005

Description - The table presents the accuracy data of radiologists compared to radiologists + Artificial Intelligence, as studied in the respective articles; AUC - Area Under the ROC Curve; NR - not reported; *This study compared AI without a radiologist (AUC 0.852) vs AI with a radiologist (AUC 0.935), showing a variation of AUC 0.083; Radiologist without AI wasn’t reported

Al-Bazzaz et al.^30^ and Elhakim et al.^32^compared the sensitivity and specificity of radiologists without AI compared to radiologists with AI, reporting no AUC. Al-Bazzaz et al.^30^ demonstrated greater specificity when associated with AI (with AI: 0.85 vs. without AI: 0.67; Δ 0.28; p < 0.001), while sensitivity was statistically lower compared to the radiologist without the aid of AI (with AI: 0.78 vs. without AI: 0.84; Δ -0.06; p = 0.017). While Elhakim et al.^32^ reported a non-statistically higher sensitivity when associated with AI (with AI: 0.746 vs. without AI: 0.739; Δ 0.007; p = 0.32), and a statistically non-inferior specificity (with AI: 0.973 vs. without AI: 0.979; Δ -0.006; p < 0.0001). False-negative rates were observed to decrease when AI algorithms were added to radiologists’ evaluation of mammograms. Elhakim et al.,^32^Kim et al.^24^ and Pacilè et al.^25^reported reductions of 8,6%, 11%, and 18%, respectively. The recall rates decreased because there was less need for additional evaluation because of potential malignancy suspicion as a result of the decline in false negatives.

## Discussion

To improve the examination’s accuracy compared to single reading, which is mainly utilized in Brazil, many nations, including the United States and European countries, have adopted double-reading mammography screening. Artificial intelligence (AI) has become a tool that can be independently used in mammographic screening in this context, effectively taking on the role of the first reader, as these algorithms show performance comparable to radiologists (as evidenced by the studies presented in this systematic review). Additionally, AI has emerged as an effective way of addressing the global shortage of radiologists as well as minimizing errors and incorrect findings reported by medical professionals.^16^

The workload of radiologists can be reduced and evaluation time saved by using artificial intelligence as the first reader for mammograms that could have been interpreted by the algorithm. According to studies, the workload reduction rate can range from 60% and higher. In this way, radiologists could perform a greater number of mammograms with more specific precision to identify findings that could go unnoticed, such as interval breast cancer.^17,26,30^

Combining AI with radiologist assessment is an ideal approach to advance this technology in the field in countries where mammography studies are performed by a single professional. The accuracy of the assessment and the precision of the examination are enhanced due to this combination’s capacity to increase sensitivity and AUC. In terms of evaluation time, it was not claimed to differ significantly from the use of Deep Learning algorithms.^4,10^

It’s important to bring attention to the studies’ limitations, including the use of algorithms that were developed for one population and then applied to another, relatively small samples with no statistical power, enrichment of positive diagnoses, and selection biases that are common in retrospective studies. The use of various artificial intelligence algorithms in the studies can be a limitation, as some algorithms may be more accurate for certain findings or specific ethnic groups. Therefore, it is essential to evaluate each type of algorithm individually to determine its suitability for that population.

About the evaluation of mammograms from two different populations (the United Kingdom and Hungary), Sharma et al.^19^ reported differing sensitivities of the algorithm, and Hsu et al.^27^ also reported this variation depending on the ethnicity of the women. When comparing age and breast density subgroups, differences in algorithm accuracy were also demonstrated, with the algorithm being less accurate in women under 50 and those with dense breasts.^11^

In general, AI algorithms used in breast cancer screening have come to be considered an instrument to help improve the efficiency and precision of radiologists. They also help less experienced doctors to identify potentially cancerous lesions. These algorithms can also be useful tools for screening, minimizing workload, and lowering unnecessary recall rates in institutions where assessments involve two readers.

However, you have to be aware of the tendency to follow an erroneous AI suggestion, when you trust the AI system too much, especially those less experienced radiologists, who end up making changes to up to 48% of mammograms after findings provided by artificial intelligence, according to the report provided by the study by Al-Bazzaz et al.,^30^however, these findings are not always true, leading to an error in reading the mammogram.

Deep Learning algorithms for evaluating breast and particular lesions, however, still require further study. This is demonstrated by the work of Liu et al.,^28^who assessed an AI model to identify malignancy in patients with microcalcifications (p = 0.029). The model performed better at identifying malignancy than inexperienced radiologists. Another critical area for future research is the creation of AI systems that can assess mammograms based on the patient’s age and ethnicity.

Therefore, prospective randomized multicenter studies comparing AI models vs. radiologists without AI are needed for external validation of such tools in the cancer screening setting. To compare such groups in the real clinical world, without being subject to the common biases of prospective studies. In addition to presenting high statistical power to confirm or discard the hypothesis that AI has equal or better accuracy than radiologists in evaluating mammograms.^39^

## Conclusion

Artificial intelligence has been demonstrated to be an effective instrument for additional evaluation in the screening for breast cancer, either as the initial reader or as a resource for radiologists. These conclusions are based on studies included in this work that showed accuracy and precision comparable to or superior to those of experienced radiologists. As a result, these algorithms are useful tools that can be incorporated into the daily operations of mammography centers as a replacement for the first reader in dual-read locations, or associated with the radiologist in single-read countries. However, because different sensitivities have been reported in diverse populations, it is essential to develop AI tailored to particular populations and ethnicities. In addition, prospective studies are needed that externally validate the algorithms in the real world.
